# Mild Adrenal Steroidogenic Defects and ACTH-Dependent Aldosterone Secretion in High Blood Pressure: Preliminary Evidence

**DOI:** 10.1155/2014/295724

**Published:** 2014-12-15

**Authors:** João Martin Martins, Sónia do Vale, Ana Filipa Martins

**Affiliations:** ^1^Endocrine Department, Hospital Santa Maria and Lisbon Medical School, Professor Egas Moniz Avenue, 1649-028 Lisbon, Portugal; ^2^Serviço de Endocrinologia, Hospital de Santa Maria, Piso 6, Avenida Professor Egas Moniz, 1649-028 Lisboa, Portugal

## Abstract

*Introduction*. Adrenal glands play a major role in the control of blood pressure and mild defects of steroidogenesis and/or inappropriate control of mineralocorticoid production have been reported in high blood pressure (HBP). *Patients and Methods*. We used a specific protocol for the evaluation of 100 consecutive patients with inappropriate or recent onset HBP. Specific methods were used to confirm HBP and to diagnose secondary forms of HBP. In addition we tested adrenal steroidogenesis with the common cosyntropin test, modified to include the simultaneous measurement of renin and aldosterone besides 17-hydroxyprogesterone (17OHP) and 11-deoxycortisol (S). *Results*. Secondary forms of HBP were diagnosed in 32 patients, including 14 patients with primary hyperaldosteronism (PA) (14%) and 10 patients with pheochromocytoma (10%). Mild defects of the 21-hydroxylase (21OHD) and 11-hydroxylase (11OHD) enzymes were common (42%). ACTH-dependent aldosterone secretion was found in most patients (54%) and characteristically in those with mild defects of adrenal steroidogenesis (>60%), PA (>75%), and otherwise in patients with apparent essential HBP (EHBP) (32%). *Discussion*. Mild defects of adrenal steroidogenesis are common in patients with HBP, occurring in almost half of the patients. In those patients as well as in patients with apparent EHBP, aldosterone secretion is commonly dependent on ACTH.

## 1. Introduction

HBP occurs in more than 25% of the adult western population and is a major determinant of mortality, with cardio- and cerebrovascular disease accounting for 30–50% of all deaths [1].

Until recently, HBP was assumed to be idiopathic in over 90% of the cases, and the search for the etiology was a futile clinical exercise in most instances [2]. As an example of changing medical paradigms, secondary forms of HBP, mainly PA, are now considered to account for up to 15% of all cases [3].

Adrenal glands play a major direct role in water and salt balance, mineralocorticoids and glucocorticoids, and in heart output and vascular tone, catecholamines; they are therefore a major determinant of blood pressure levels in normal conditions [3–5]. Specific adrenal diseases like PA, Cushing's syndrome, pheochromocytoma, and some cases of congenital adrenal hyperplasia (CAH) and glucocorticoid-remediable aldosteronism (GRA) are well recognized forms of secondary HBP [1–7]; however, except for PA, all are deemed to be very rare.

A more general involvement of the adrenals may occur in HBP. Recent research identifies mild defects of the final steps of glucocorticoid and/or mineralocorticoid synthesis, namely, defects of the 11*β*-hydroxylase enzyme and abnormal control of aldosterone secretion, ACTH-dependent aldosterone secretion, as common and related phenomena in patients with HBP [8–12]. We tested adrenal steroidogenesis and mineralocorticoid production, using the common cosyntropin test, modified to include the measurement of renin and aldosterone, besides cortisol, 17OHP, and S, in patients with recent onset or inappropriate HBP, as defined by Kaplan [2].

## 2. Patients and Methods

We selected, from the Outpatient Endocrine Clinic, patients with inappropriate HBP as defined by Kaplan [2], that is, (a) HBP beginning before 20 or after 50 years; (b) HBP with very high levels to begin with, with early evidence of target organs lesions, or requiring from the beginning several antihypertensive drugs to control blood pressure levels; (c) HBP with special clinical clues suggesting a secondary form, including hypokalemia, hypertensive crisis, and vascular bruits over the renal areas; (d) family history of early onset HBP.

Etiologic factors of HBP may be apparent only during the early stages of the disease, since with time HBP tends to become fixed and dependent on increased peripheral vascular resistance [2]. We also included patients with recent onset HBP, defined as less than 5 years since diagnosis.

These patients were admitted to the Endocrine Inpatient Department after at least a weekend with only Nifedipine used to treat HBP or at least three weeks after suspending spironolactone use and entered into a specific evaluation protocol: (1) baseline, including serum glucose, urea, creatinine, ionogram, calcium, phosphate, magnesium, thyroid and parathyroid function, insulin, ACTH, cortisol, renin, aldosterone, urinary catecholamines, metanephrine and normetanephrine, EKG, chest radiograph, echocardiogram, renal sonography, and the 24 h measurement of blood pressure levels (24HBP); (2) the saline suppression test, 2 L of normal saline, iv, in four hours with measurement of renin and aldosterone before and after the procedure; (3) the corticotropin releasing hormone test measuring ACTH and cortisol before and at 5, 10, 15, 30, 60, and 120 min after the iv administration of corticotropin releasing hormone 100 *μ*g; (3) the clonidine test with measurement of serum catecholamines, metanephrines, and normetanephrines, before and 3 h after the administration of 300 mg of clonidine po; (4) oral tolerance test with 75 g of glucose and measurement of serum glucose and insulin at times 0, 30, 60, 90, and 120 min; (5) personality evaluation using the Minnesota Multiphase Personality Inventory (MMPI). A modified version of the common cosyntropin test was also administered: 250 *μ*g of cosyntropin (1-24 ACTH) was administered iv, at time 0, and measurement before and at 30 and 60 min after of cortisol, 17OHP, S, renin, and aldosterone was obtained.

This protocol was modified according to the specific clinical characteristics of the patients, and therefore not all tests were done in every patient, while some specific tests like the furosemide and the captopril tests or the dexamethasone suppression test were sometimes performed. Imaging methods followed as appropriate.

All serum measurements were performed in duplicate at the Clinical Pathology Department using standard methodology and commercially available kits. More specifically radioimmunoassay (RIA) was used for the measurement of 17OHP (Coat-A-Count 17*α*-OH Progesterone, Siemens), aldosterone (Coat-A-Count Aldosterone, Siemens), and S (DIAsource 11-deoxycortisol-RIA-CT), an immunoradiometric assay (IRMA) for renin (DIAsource Renin-IRMA), while for cortisol measurement a colorimetric enzyme-linked immunoassay was used (CLIA) (Bayer Diagnostics ADVIA Centaur). Intra- and interassay variation were always <10%, and regarding 17OHP and S assays, cross reactivity was <6%. Reference baseline decubitus values are as follows: cortisol 3–23 *μ*g/dL, 17OHP 0–5 ng/mL, S 0–5 ng/mL, renin 1–20 *μ*U/mL, and aldosterone 10–160 pg/mL.

The statistical package for the Social Sciences software (IBM SPSS19, New York) was used for the analysis. Results are expressed as the mean ± standard deviation (*μ* ± *σ*) or as % as appropriate. The normal distribution of the continuous variables was verified with the Kolmogorov-Smirnov test (KS), or the log-transformed variables were used; however when no difference was found, results regarding the nontransformed variable are presented. The Chi-squared test, the Student *t* test, or factorial ANOVA was used to compare groups and regression analysis to explore the relation between continuous variables.

## 3. Results

**Figure 1:**
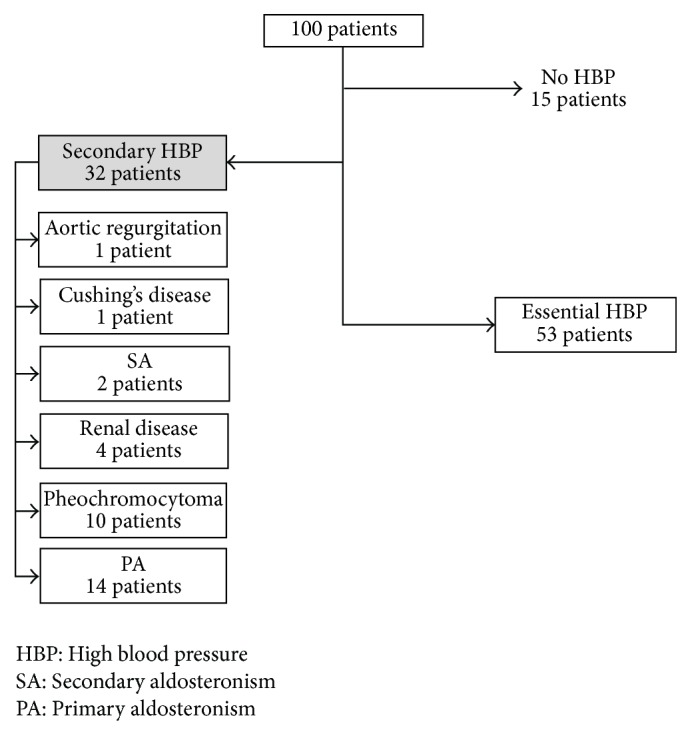
Diagnostic workup of the patients. HBP, high blood pressure; PA, primary aldosteronism; SA, secondary aldosteronism.

We studied 112 consecutive patients. However for several reasons, the protocol was too incomplete to be useful in 12 patients and we now report the results of 100 patients. Patients were mainly female (66%) and middle-aged (46 ± 15 years). HBP had been diagnosed 8 ± 8 years before. Results of the diagnostic workup of the patients are presented in Figure 1.

In fifteen patients (15%) the diagnosis of HBP was specifically excluded. In hospital and afterwards during usual life, 24HBP without any medication was normal, with no evidence of target organ lesions.

A secondary form of HBP was diagnosed in 32 cases (32%). There were fourteen (14%) cases of PA, increased aldosterone/renin ratio, no suppression of aldosterone levels in the saline test, and from these 7 were adenoma, single nodular lesion in the adrenal with a positive ^131^I-iodomethylnorcholesterol (^131^IMNC) scan and decreased aldosterone after 4 h of deambulation, and 7 were bilateral hyperplasia, no nodular lesion in the CT scan of the adrenals, bilateral hyperfixation on the (^131^NIMC) scan, and increased aldosterone after 4 h deambulation. In ten cases (10%) the diagnosis of pheochromocytoma was established, increased urinary metanephrine and/or normetanephrine levels, no suppression of metanephrine and/or normetanephrine levels in the clonidine test, a nodular lesion on the adrenals CT scan, and a positive ^123^I-metaiodobenzylguanidine (^123^MIBG) scan. There were four cases (4%) of HBP secondary to renal disease, renal disease with increased serum creatinine levels, two cases (2%) of secondary hyperaldosteronism (SA), one with documented renal stenosis and the other probably in relation to contraceptive use, one case (1%) of Cushing's disease with no suppression of cortisol levels after prolonged low dose dexamethasone, suppressed cortisol after prolonged high dose dexamethasone and a pituitary lesion on sellar NMR, and one case (1%) of isolated systolic HBP with documented aortic regurgitation.

Essential HBP (EHBP) was diagnosed in the remaining 53 cases (53%). However even in these patients one or more presumptive contributory mechanisms for HBP could be identified in all but nine cases, which guided therapy selection: obesity (26 patients), hyperinsulinism (7 patients), type 2 diabetes (18 patients), chronic stress (11 patients), contraceptive use (2 patients), and possible reaction to antidepressive drugs (3 patients).

The cosyntropin test was not performed in the 15 patients with no HBP, neither on the 10 patients with a diagnosis of pheochromocytoma nor on 10 patients with apparent EHBP. Therefore the test was only preformed in 65 cases. Results of the cosyntropin test are presented in Table 1.

**Figure 2:**
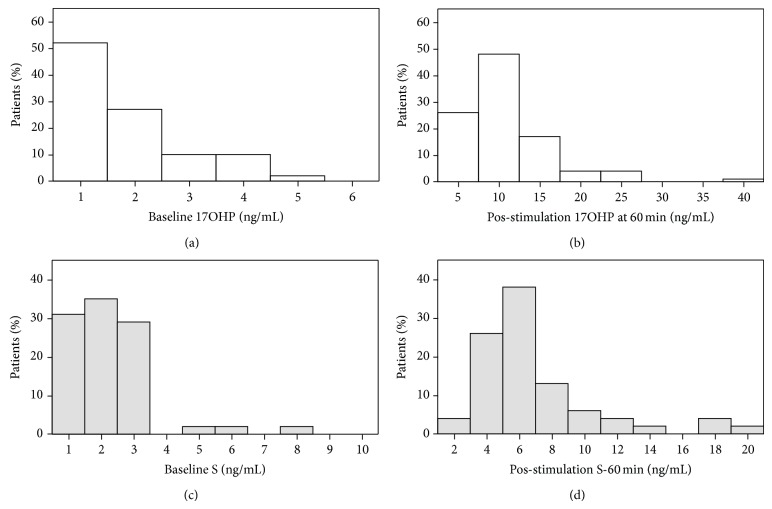
(a) Histogram of baseline 17-hydroxyprogesterone (17OHP) distribution. (b) Histogram of 17-hydroxyprogesterone (17OHP) distribution at 60 min after 250 *μ*g of cosyntropin iv. (c) Histogram of baseline 11-deoxycortisol (S) distribution. (d) Histogram of 11-deoxycortisol (S) distribution at 60 min after 250 *μ*g of cosyntropin iv.

The distribution of 17OHP and S values at baseline and pos-stimulation at 60 min are presented in Figure 2. The relation between skewness and kurtosis suggests that the baseline 17OHP distribution is unimodal, while the same is not true regarding the distributions of baseline S or pos-stimulated levels of either 17OHP or S [13]. Besides, baseline and pos-stimulation levels of 17OHP and S are not normally distributed (K-S z between 1.341 and 2.740, *P* < 0.05). Glucocorticoid steroidogenesis was intrinsically related that is, baseline cortisol was directly related to 17OHP (*r* = 0.388, *P* < 0.05) and to S (*r* = 0.329, *P* < 0.05), while this was not true regarding mineralocorticoid steroidogenesis, no significant relation of renin and aldosterone, and neither were glucocorticoid and mineralocorticoid steroidogenesis related, no significant relation of baseline renin or aldosterone with cortisol, 17OHP or S.

Criteria for the 21-hydroxylase defect (21OHD) are well defined and include a 17OHP response equal to or greater than 10 ng/mL in the cosyntropin test [14, 15]. Although in every case baseline levels were within the reference range, using this criterium, 10 out of 65 patients (15%) at 30 min and 17 out of 65 patients (26%) at 60 min presented preliminary evidence for a mild defect of the 21-hydroxylase enzyme. Taken together 21 out 65 patients (32%) presented such evidence. As noted this defect was not apparent at baseline levels. In fact baseline 17OHP levels are only weakly related to pos-stimulated 30 min levels (*r* = 0.232, *P* < 0.1) or to pos-stimulated 60 min levels (*r* = 0.228, *P* < 0.1) while pos-stimulated 30 and 60 min levels are strongly related (*r* = 0.978, *P* < 0.001).

Criteria for the 11-hydroxylase defect (11OHD) are less well defined, but most authors indicate an S response equal to or greater than 8 ng/mL in the same test [15, 16]. Since the baseline or pos-stimulated ratio of S/17OHP is always close to 1 [15], we more strictly defined such a defect as a response greater than 10 ng/mL. Only 3 patients presented slightly increased baseline levels. Using that criterium however, 7 out of 65 patients (11%) at 30 min, 11 out of 65 patients (17%) at 60 min, and taken together 14 out of 65 patients (22%) presented preliminary evidence for a mild defect of the 11-hydroxylase enzyme. Baseline levels were related to pos-stimulation levels, at 30 min (*r* = 0.467, *P* < 0.001) and at 60 min (*r* = 0.429, *P* < 0.005), while pos-stimulated levels were strongly related to each other (*r* = 0.926, *P* < 0.001).

A defect of the 11-hydroxylase enzyme may also result in increased 17OHP levels, besides increased S levels; in fact this occurs in 8 out of the 14 patients (57%) with a postulated 11OHD; if we therefore exclude such patients from the category of 21OHD, we find that 14 out of 65 patients (22%) presented evidence for a mild 11OHD and 13 out of 65 (20%) presented evidence for a mild 21OHD.

Regarding the 13 patients with apparent mild 21OHD most patients presented EHBP, 12 patients, but it was also found in one patient with PA-hyperplasia, so that the 21OHD is found in 28% of the patients with EHBP (12 out of 43). Regarding the 14 patients with apparent mild 11OHD most patients presented EHBP, 9 patients, but it was also found in 2 patients with PA-adenoma and in 1 patient with PA-hyperplasia and in 2 patients with HBP secondary to renal disease, so that the 11OHD is found in 20% of the patients with EHBP (9 out of 43) and in 21% of PA (3 out 14).

To emphasize the differences, characteristics of steroidogenesis in patients without defect, with the 21OHD and with the 11OHD, are indicated in Table 2.

For patients with apparent mild 21OHD, (1) the evidence for the defect is given by the increased 17OHP response and because of a much decreased cortisol/17OHP ratio at 60 min, that is, much decreased cortisol production in relation to 17OHP; (2) interestingly enough these patients present slightly but significantly increased pos-stimulated S levels, albeit to a much lesser degree than patients with the 11OHD.

For patients with the apparent mild 11OHD, (1) the defect is evident by the increased S response and accessorily because of higher baseline levels of S and a much decreased cortisol/S ratio at 60 min, that is, much less cortisol produced in relation to S; (2) these patients with the 11-hydroxylase defect also present evidence of the 21-hydroxylase defect, and this is not unexpected; that is, a distal defect does result in a proximal increase of precursor compounds, like 17OHP.

Baseline and pos-stimulated levels of either cortisol, renin, or aldosterone were not significantly different regarding patients with or without postulated defects of steroidogenesis.

As noted in Table 1, a marked aldosterone response was found in the cosyntropin test. In fact the peak/baseline ratio for aldosterone is even superior to that for cortisol. Also as noted in Table 1, this response is clearly not renin dependent, since no major change is found regarding renin levels and in fact, renin levels at baseline, at 30 min and at 60 min are not significantly different. The seemingly inescapable conclusion is therefore that ACTH (or at least the synthetic analog 1-24 ACTH) is the relevant stimulus for the aldosterone response. Interestingly enough baseline levels of renin and aldosterone were not significantly related.

Using an arbitrary criterium, an aldosterone response greater than that of cortisol at 60 min, that is, an aldosterone at 60 min/baseline aldosterone > cortisol at 60 min/baseline cortisol, 35 out of 65 patients were found (54%) to present an aldosterone response to ACTH greater than that of cortisol, pointing to the relevance of ACTH in aldosterone secretion. This “abnormal” aldosterone response is found in 64% of the patients with evidence for the 11*β*-hydroxylase defect (9 out of 14), in 62% of the patients with evidence for the 21-hydroxylase defect (8 out of 13), in 86% of the patients with PA-hyperplasia (6 out 7), in 71% of the patients with PA-adenoma (5 out of 7), and in 100% of the patients with HBP secondary to renal disease (4 out of 4). Excluding these specific groups, the “abnormal” ACTH-dependent aldosterone response is still found in 32% of the patients with recent onset or inappropriate HBP (7 out of 22).

**Figure 3:**
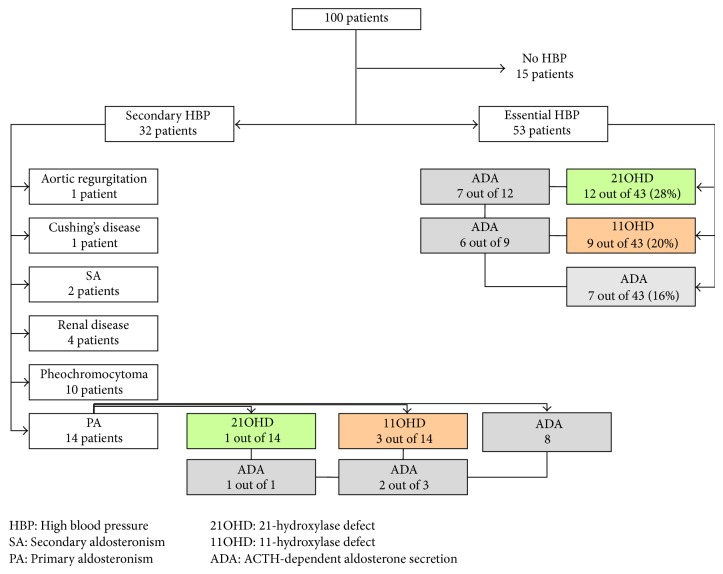
Abnormal steroidogenesis in patients with essential high blood pressure and in those with primary hyperaldosteronism. ADA, ACTH-dependent aldosterone secretion; HBP, high blood pressure; PA, primary aldosteronism; SA, secondary aldosteronism; 21OHD, 21-hydroxylase defect; 11OHD, 11-hydroxylase defect.

In summary we found that mild defects of the late stages of adrenal steroidogenesis, involving the 21-hydroxylase and the 11*β*-hydroxylase enzymes, are very common in PA (28%) and in EHBP (48%); these defects are characteristically associated with an abnormal resetting of mineralocorticoid production now depending on ACTH (>60%), which otherwise even in the absence of apparent defects of adrenal steroidogenesis is also very common in PA (57%) and in EHBP (32%). At least one of these “abnormalities” is found in 79% of patients with PA and in 65% of patients with apparent EHBP. These data are summarized in Figure 3, excluding the small number of patients with renal disease.

## 4. Discussion

Adrenal glands play a major role in the homeostasis of blood pressure levels. Several specific adrenal diseases are known as causes of secondary HBP, the most common one being PA [1–3]. However a more general involvement of adrenal steroidogenesis, mainly regarding mineralocorticoid production and/or control, has been suggested in EHBP [8–12].

This paper reports three major findings. (1) Mild defects of the 21-hydroxylase and the 11-hydroxylase enzymes are very common in inappropriate or recent onset HBP, even if theoretically the first one is not mechanistically associated with HBP. (2) Strong ACTH- dependence of aldosterone is very common in the context of inappropriate HBP, either EHBP or secondary forms of HBP, namely, PA and renal disease. (3) This ACTH-dependence of aldosterone is characteristic of mild defects of adrenal steroidogenesis, either 21-hydroxylase or 11-hydroxylase defects, being found in almost two-thirds of such cases. The inappropriate resetting of mineralocorticoid production and secretion may be a relevant pathogenic mechanism for HBP and may explain the unexpected association of 21-hydroxylase defects with HBP.

We first report a high prevalence (22%) of apparent mild 11OHD in selected patients with inappropriate HBP. We used a more restricted criterium of an S response greater than 10 ng/mL to cosyntropin, the same quantitative criterium that is generally accepted for the more common 21OHD regarding the 17OHP response. This restriction seems justified since 17OH/S ratios are always near to or greater than 1 [15, 16]. Several lines of evidence suggest that this mild defect is real: (a) baseline S levels and stimulated S response to cosyntropin do not fit a unimodal distribution and are not normally distributed; (b) baseline values of S are higher than in the group without the defect; (c) the stimulated cortisol/S ratio is significantly lower suggesting indeed the decreased effectiveness of 11-deoxycortisol to cortisol conversion; (d) as shown patients with this mild apparent defect characteristically present an ACTH-dependent aldosterone response (64%).

Of course this is not a major defect [4–6, 16–18]. The classic 11OHD is a rather rare condition, occurring in only 1 : 20,000 live births and accounting for only 8% of all cases of CAH. Furthermore the classic form presents with sexual ambiguity, advanced growth albeit with limitation of final height, precocious puberty, oligomenorrhea, and hirsutism. Much more probably this mild defect that is only apparent after supraphysiological stimulation corresponds to either simple heterozygotes, which may be up to 100 times more common although it is controversial whether such patients present any endocrine abnormalities [19], compound heterozygotes, or common polymorphisms of the* CYP11B1* gene [11, 12, 20, 21]. It may even correspond to a secondary acquired defect, although we are not aware of known modulators of this enzyme activity besides glycyrrhizin acid or metyrapone [3–7].

Genetic studies as well as measurement of specific steroid compounds, like 11-deoxy corticosterone (DOC) and corticosterone, are necessary to further characterize these defects, although very large samples will probably be needed given both the mild nature of the defect and the probable genetic heterogeneity.

Other authors have previously reported this mild defect in patients with HBP and in patients with PA [8, 10–12, 20, 21]. Isolated reports also point to the association of this mild defect with an apparent ACTH-dependent aldosterone secretion [9]. Our data suggest that this is indeed a very common association (66%). It should be noted that both the increased levels of precursors with mineralocorticoid activity, that is, deoxy corticosterone and the abnormal resetting of mineralocorticoid production by ACTH, could contribute to HBP.

We also report on an unexpectedly high frequency of mild 21OHD (20%). An increased 17OHP response is found in more patients, but these include patients with mild defects of 11OHD which is of course not unexpected. It emphasizes the need of the simultaneous measurement of S and 17OHP to define 21OHD and may contribute to the reportedly low frequency of the 11OHD; should only 17OH be measured, many 11OHD will instead be classified as 21OHD [4–6, 14]. Again the same arguments may be made regarding the validity of that defect, namely, the nonunimodality and nonnormality of the 17OHP response, the decreased cortisol/17OH ratio, and, up to a point, the ACTH-dependent aldosterone response. Again, and for the same reasons as before, this is not a major defect and most probably corresponds either to heterozygotes or to polymorphisms of the CYP21 gene, even if an acquired defect cannot be excluded [4–6, 13, 21].

Two additional points characterize patients with apparent mild 21OHD. In such a defect, S levels should be expected to be decreased; instead in these patients, and regarding patients without apparent defects of the steroidogenesis, S levels are increased, albeit not to the level of that found in patients with 11OHD. This suggests that these patients also present a defective conversion of S to cortisol that is not more evident given the presence of the 21OHD [14, 22–24]. Secondly, these patients also present an ACTH-dependent aldosterone response that so far has not been previously reported, except in single case reports [25]. Both the double block, involving the 11-hydroxylase enzyme besides the 21-hydroxylase enzyme, and the ACTH-dependent aldosterone response could explain the otherwise paradoxical association of the 21OHD with HBP. Interestingly enough, some recent reports suggest increased systolic and/or diastolic blood pressure levels in patients 21-hydroxylase deficiency with systolic but not diastolic blood pressure levels being related to body mass index, and neither being related to glucocorticoid doses [26, 27].

The large frequency of 21OHD and 11OHD argues against a genetic background; instead an adaptive response is suggested. Teleologically, limitation of adrenal steroidogenesis, by mild blockade at the 21-hydroxylase and 11*β*-hydroxylase enzymes, could be of value, in the context of HBP by limiting final glucocorticoid and mineralocorticoid production. However, as noted below, ACTH-dependent aldosterone production could offset the protective effect of such a response.

Regarding patients with apparent mild 21-hydroxylase and/or 11-hydroxylase defects, data regarding 24 h urinary sodium and potassium excretion may be informative. Obtaining that data might require however strict control of dietary ingestion or very large samples to allow for a meaningful interpretation.

The third major finding was that an extremely common ACTH-dependent aldosterone secretion is operatively defined as an aldosterone response greater than that of cortisol in the cosyntropin test. This is new in the sense that ACTH is generally assumed to be a minor factor controlling aldosterone secretion, contrary to what happens in regard to cortisol, since aldosterone secretion is mainly dependent on the renin-angiotensin axis [4, 5, 28]. As noted the response on the cosyntropin test clearly does not depend on renin. We suppose that this is another way to look at the same phenomenon of aldosterone suppression and HBP correction by dexamethasone that is generally used to define GRA [29]. The molecular basis of that difference resides in the promoter of the* CYP11B1* gene, with responsive elements for ACTH and that of the* CYP11B2* gene, with elements responsive to renin. Unequal crossing over between adjacent* CYP11B1 *and* CYP11B2* genes that are very close on chromosome 8q21, 40 kb apart, where the promoter regulatory region of the CYP11B1 gene is juxtaposed to the coding region of the* CYP11B2* gene, results in a chimeric enzyme, overexpressed in the fasciculata zone, under the control of ACTH and to aldosterone excess and HBP [30, 31]. However as described that is a very rare disease with a completely different presentation, that is, familial HBP, beginning very early in life and with a severe course and early end organ damage [32–34]. Again this is not probably what we found. However it should be noted that ACTH-dependence of aldosterone secretion has been noted without such genetic abnormalities [35], while it has been also reported in patients with 11-hydroxylase defects [8–10, 12, 21] and is characteristic of PA-adenoma [3] and it is extremely unlikely that both those conditions would also present that genetic defect. It seems more likely that ACTH-dependent aldosterone secretion, as defined, results from convergent signaling pathways from angiotensin II and ACTH, respectively, phospholipase C and adenylyl cyclase [36]. Cross-talk between these two activating systems may be modulated by several cellular signals as shown recently regarding Sprouty-related protein with EVH1 domain 2 [37].

In this report we found that ACTH-dependent aldosterone secretion is very common in EHBP or at least in the highly selected group of the patients such as this one but furthermore characterizes patients with disordered steroidogenesis, 11-hydroxylase and 21-hydroxylase defects, patients with PA either hyperplasia or adenoma, and patients with HBP secondary to renal diseases. Of course such a general aldosterone response to ACTH strongly argues against such a defect; on the contrary it suggests some kind of regulatory effect that makes the* CYP11B2* gene responsive to ACTH. Other authors have suggested the same possibility [8–12]. However the reason/mechanism for that response in the other cases is unknown. Interestingly enough, both the* CYP11B1* and the* CYP11B2* genes respond to intracellular AMPc [3–5]. It seems therefore possible that mild defects of steroidogenesis with the compensatory increased drive of ACTH could result in a ACTH-dependent aldosterone secretion. ACTH may also, in some cases at least, be the stimulus driving PA-hyperplasia that may sometimes evolve to adenoma [24]. It has been suggested that in a significant group of hypertensive patients, probably up to 15% with low renin levels, aldosterone may indeed be ACTH-responsive [11, 12]. Whatever the reason, however, a* CYP11B2* responsive to ACTH would theoretically, as in GRA, lead to inappropriate aldosterone levels and HBP.

## Figures and Tables

**Table 1:** Cosyntropin (1–24 ACTH) test (250 mg, iv at time 0).

	0 min	30 min	60 min
Cortisol [3–23 *µ*g/dL]	16 ± 7 [6–48]	38 ± 11 [20–51]	39 ± 10 [10–51]
17OHP [0–5 ng/mL]	2 ± 1 [1–5]	8 ± 6 [1–39]	8 ± 6 [1–38]
S [0–2 ng/mL]	2 ± 1 [1–8]	6 ± 4 [2–25]	7 ± 4 [2–19]
Renin [1–20 *µ*U/mL]	10 ± 20 [1–149]	11 ± 21 [1–142]	11 ± 18 [1–121]
Aldosterone [10–160 pg/mL]	114 ± 171 [2–859]	221 ± 313 [10–1,628]	334 ± 522 [5–3,656]

Conversion factors are as follows: cortisol *µ*g/dL × 27.59 = cortisol nmol/L; 17OHP ng/mL × 3.026 = 17OHP nmol/L; S ng/mL × 2.887 = S nmol/L; aldosterone pg/mL × 0.002774 = aldosterone nmol/L.

**Table 2:** Steroidogenesis in patients with no defect (A) or with the 21OHD (B) or 11OHD (C).

	No defect (A) (38)	21OHD (B) (13)	11OHD (C) (14)
17OHP baseline (ng/mL)	2 ± 1	2 ± 1	3 ± 13^c^
17OHP 30 min (ng/mL)	5 ± 2	10 ± 1^a,c^	15 ± 11^b,c^
17OHP 60 min (ng/mL)	5 ± 2	11 ± 2^a,c^	15 ± 11^b,c^
C17OHP 60 min^1^	8.7 ± 5.4	4.3 ± 1.6^a^	3.2 ± 1.8^b^

S baseline (ng/mL)	2 ± 1	2 ± 1	4 ± 2^b^
S 30 min (ng/mL)	4 ± 1	6 ± 2^a,c^	12 ± 6^b,c^
S 60 min (ng/mL)	5 ± 2	6 ± 1^a,c^	14 ± 3^b,c^
CS 60 min^2^	8.1 ± 3.0	7.0 ± 2.6^c^	3.0 ± 0.5^b,c^

^1^C17OHP 60 min cortisol/17OHP ratio at 60 min, arbitrary units; ^2^CS 60 min cortisol/S ratio at 60 min, arbitrary units; ^a^significant differences between groups B and A; ^b^significant differences between groups C and A; ^c^significant differences between groups C and B; in every case post hoc analysis when analysis of variance between the three groups revealed significant differences.

## References

[1] Mancia G., Fagard R., Narkiewicz K. (2013). 2013 ESH/ESC Guidelines for the management of arterial hypertension. The Task Force for the management of arterial hypertension of the European Society of Hypertension (ESH) and of the European Society of Cardiology (ESC). *Journal of Hypertension*.

[2] Kaplan N. M. (2006). *Kaplan’s Clinical Hypertension*.

[3] Young W. F., Melmed S., Polonsky K. S., Larsen P. R., Kronenberg H. M. (2011). Endocrine hypertension. *Williams Textbook of Endocrinology*.

[4] Stewart P. M., Krone N. P., Melmed S., Polonsky K. S., Larsen P. R., Kronenberg H. M. (2011). The adrenal cortex. *Williams Textbook of Endocrinology*.

[5] Stewart P. M., Kronenberg H. M., Melmed S., Polonsky K. S., Larsen P. R. (2008). The adrenal cortex. *Williams Textbook of Endocrinology*.

[6] Wajnrajch M. P., New M. I., Jameson J. L., de Groot L. J. (2010). Defects of adrenal steroidogenesis. *Endocrinology Adult and Pediatric*.

[7] Carey R. M., Padia S. H., Jameson J. L., de Groot L. J. (2010). Primary mineralocorticoid excess syndromes and hypertension. *Endocrinology Adult and Pediatric*.

[8] de Simone G., Tommaselli A. P., Rossi R., Valentino R., Lauria R., Scopacasa F., Lombardi G. (1985). Partial deficiency of adrenal 11-hydroxylase: a possible cause of primary hypertension. *Hypertension*.

[9] Jamieson A., Ingram M. C., Inglis G. C., Davies E., Fraser R., Connell J. M. C. (1996). Altered 11*β*-hydroxylase activity in glucocorticoid-suppressible hyperaldosteronism. *Journal of Clinical Endocrinology and Metabolism*.

[10] Connell J. M. C., Fraser R., MacKenzie S., Davies E. (2003). Is altered adrenal steroid biosynthesis a key intermediate phenotype in hypertension?. *Hypertension*.

[11] Imrie H., Freel M., Mayosi B. M., Davies E., Fraser R., Ingram M., Cordell H. J., Farrall M., Avery P. J., Watkins H., Keavney B., Connell J. M. C. (2006). Association between aldosterone production and variation in the 11*β*-hydroxylase (CYP11B1) gene. *Journal of Clinical Endocrinology and Metabolism*.

[12] Alvarez-Madrazo S., Padmanabhan S., Mayosi B. M. (2009). Familial and phenotypic associations of the aldosterone renin ratio. *The Journal of Clinical Endocrinology & Metabolism*.

[13] Klaassen C. A. J., Mokveld P. J., van Es B. (2000). Squared skewness minus kurtosis bounded by 186/125 for unimodal distributions. *Statistics and Probability Letters*.

[14] Speiser P. W., Azziz R., Baskin L. S., Ghizzoni L., Hensle T. W., Merke D. P., Meyer-Bahlburg H. F. L., Miller W. L., Montori V. M., Oberfield S. E., Ritzen M., White P. C. (2010). Congenital adrenal hyperplasia due to steroid 21-hydroxylase deficiency: an endocrine society clinical practice guideline. *The Journal of Clinical Endocrinology and Metabolism*.

[15] (1991). *Adrenal Steroid Response to ACTH: Pediatrics*.

[16] Parajes S., Loidi L., Reisch N., Dhir V., Rose I. T., Hampel R., Quinkler M., Conway G. S., Castro-Feijóo L., Araujo-Vilar D., Pombo M., Dominguez F., Williams E. L., Cole T. R., Kirk J. M., Kaminsky E., Rumsby G., Arlt W., Krone N. (2010). Functional consequences of seven novel mutations in the CYP11B1 gene: four mutations associated with nonclassic and three mutations causing classic 11*β*-hydroxylase deficiency. *Journal of Clinical Endocrinology and Metabolism*.

[17] Krone N., Riepe F. G., Götze D., Korsch E., Rister M., Commentz J., Partsch C.-J., Grötzinger J., Peter M., Sippell W. G. (2005). Congenital adrenal hyperplasia due to 11-hydroxylase deficiency: functional characterization of two novel point mutations and a three-base pair deletion in the *CYP11B1* gene. *The Journal of Clinical Endocrinology and Metabolism*.

[18] Krone N., Grischuk Y., Müller M. (2006). Analyzing the functional and structural consequences of two point mutations (P94L and A368D) in the *CYP11B1* gene causing congenital adrenal hyperplasia resulting from 11-hydroxylase deficiency. *The Journal of Clinical Endocrinology & Metabolism*.

[19] Peter M., Sippell W. G. (1997). Evidence for endocrinological abnormalities in heterozygotes for adrenal 11*β*-hydroxylase deficiency of a family with the R448H mutation in the CYP11B1 gene. *Journal of Clinical Endocrinology and Metabolism*.

[20] Ganapathipillai S., Laval G., Hoffmann I. S., Castejon A. M., Nicod J., Dick B., Frey F. J., Frey B. M., Cubeddu L. X., Ferrari P. (2005). CYP11B2-CYP11B1 haplotypes associated with decreased 11*β*-hydroxylase activity. *The Journal of Clinical Endocrinology and Metabolism*.

[21] Keavney B., Mayosi B., Gaukrodger N., Imrie H., Baker M., Fraser R., Ingram M., Watkins H., Farrall M., Davies E., Connell J. (2005). Genetic variation at the locus encompassing 11-*β* hydroxylase and aldosterone synthase accounts for heritability in cortisol precursor (11-deoxycortisol) urinary metabolite excretion. *The Journal of Clinical Endocrinology and Metabolism*.

[22] Krone N., Rose I. T., Willis D. S., Hodson J., Wild S. H., Doherty E. J., Hahner S., Parajes S., Stimson R. H., Han T. S., Carroll P. V., Conway G. S., Walker B. R., MacDonald F., Ross R. J., Arlt W. (2013). Genotype-phenotype correlation in 153 adult patients with congenital adrenal hyperplasia due to 21-hydroxylase deficiency: analysis of the United Kingdom congenital adrenal hyperplasia adult study executive (CaHASE) cohort. *Journal of Clinical Endocrinology and Metabolism*.

[23] Hurwitz A., Brautbar C., Milwidsky A., Vecsei P., Milewicz A., Navot D., Rösler A. (1985). Combined 21- and 11*β*-hydroxylase deficiency in familial congenital adrenal hyperplasia. *Journal of Clinical Endocrinology and Metabolism*.

[24] Eldar-Geva T., Hurwitz A., Vecsei P., Palti Z., Milwidsky A., Rosler A. (1990). Secondary biosynthetic defects in women with late-onset congenital adrenal hyperplasia. *The New England Journal of Medicine*.

[25] Martins J. M., Cabral R. M., do Vale S., Martins A. F., Gomes A. R. (2012). Primary hyperaldosteronism: pitfalls in the diagnosis and a not so peculiar evolution—case report. *Journal of Endocrinology and Metabolism*.

[26] Völkl T. M. K., Simm D., Dötsch J., Rascher W., Dörr H. G. (2006). Altered 24-hour blood pressure profiles in children and adolescents with classical congenital adrenal hyperplasia due to 21-hydroxylase deficiency. *The Journal of Clinical Endocrinology and Metabolism*.

[27] Nermoen I., Brønstad I., Fougner K. J., Svartberg J., Øksnes M., Husebye E. S., Løvås K. (2012). Genetic, anthropometric and metabolic features of adult norwegian patients with 21-hydroxylase deficiency. *European Journal of Endocrinology*.

[28] Oelkers W. (1985). Prolonged ACTH infusion suppresses aldosterone secretion in spite of high renin activity. *Acta Endocrinologica*.

[29] Sutherland D. J., Ruse J. L., Laidlaw J. C. (1966). Hypertension, increased aldosterone secretion and low plasma renin activity relieved by dexamethasone. *Canadian Medical Association Journal*.

[30] Lifton R. P., Dluhy R. G., Powers M., Rich G. M., Gutkin M., Fallo F., Gill J. R., Feld L., Ganguly A., Laidlaw J. C., Murnaghan D. J., Kaufman C., Stockigt J. R., Ulick S., Lalouel J.-M. (1992). Hereditary hypertension caused by chimaeric gene duplications and ectopic expression of aldosterone synthase. *Nature Genetics*.

[31] Pascoe L., Curnow K. M., Slutsker L., Connell J. M. C., Speiser P. W., New M. I., White P. C. (1992). Glucocorticoid-suppressible hyperaldosteronism results from hybrid genes created by unequal crossovers between CYP11B1 and CYP11B2. *Proceedings of the National Academy of Sciences of the United States of America*.

[32] Garovic V. D., Hilliard A. A., Turner S. T. (2006). Monogenic forms of low-renin hypertension. *Nature Clinical Practice Nephrology*.

[33] McMahon G. T., Dluby R. G. (2004). Glucocorticoid-remediable aldosteronism. *Arquivos Brasileiros de Endocrinologia & Metabologia*.

[34] Fallo F., Pilon C., Williams T. A., Sonino N., Morra Di Cella S., Veglio F., de Iasio R., Montanari P., Mulatero P. (2004). Coexistence of different phenotypes in a family with glucocorticoid-remediable aldosteronism. *Journal of Human Hypertension*.

[35] Fardella C. E., Pinto M., Mosso L., Gómez-Sánches C., Jalil J., Montero J. (2001). Genetic study of patients with dexamethasone-suppressible aldosteronism without the chimeric cyp11b1/cyp11b2 gene. *Journal of Clinical Endocrinology and Metabolism*.

[36] Spät A., Hunyady L. (2004). Control of aldosterone secretion: a model for convergence in cellular signaling pathways. *Physiological Reviews*.

[37] Ullrich M., Bundschu K., Benz P. M., Abesser M., Freudinger R., Fischer T., Ullrich J., Renné T., Walter U., Schuh K. (2011). Identification of SPRED2 (Sprouty-related protein with EVH1 domain 2) as a negative regulator of the hypothalamic-pituitary-adrenal axis. *The Journal of Biological Chemistry*.

